# The Year-Book of Treatment for 1889

**Published:** 1889-03

**Authors:** 


*The Year-book of Treatment for* 1889. Pp. 344. London: CASSELL & CO.

We gladly welcome this very useful *multum in parvo*, collecting, abstracting, and reviewing the most important medical writings *of* 1888—*for* 1889, as stated.

We have already stated our objections to abstractors being at the same time reviewers; and in this respect we have placed another medical annual ahead of the one before us. We should like to see an editor over all who was at the same time a competent grammarian, and who would ruthlessly deal with slipshod or ungrammatical English, and see that punctuation was attended to. Only a minority of the contributors sin in this respect; but their sins are very heinous. In these stirring times the following sentence comes with an effect that is at once reassuring and delicious:

"The year just past has in one sense been an uneventful one for those who are specially interested in diseases of the throat and nose."

The philosophic calm which a mental pre-occupation in nasal and throat matters would seem to beget is just what is wanted in this harassed age.

